# Supplementary material for: Genome-Wide Pathway Exploration of the Epidermidibacterium keratini EPI-7T
Source: Microorganisms. 2023 Mar 28;11(4):870. doi: 10.3390/microorganisms11040870 (PMC10143877; doi:10.3390/microorganisms11040870)
Supplement: Supplementary file 1 [file microorganisms-11-00870-s001.zip › Figure S1.pdf]

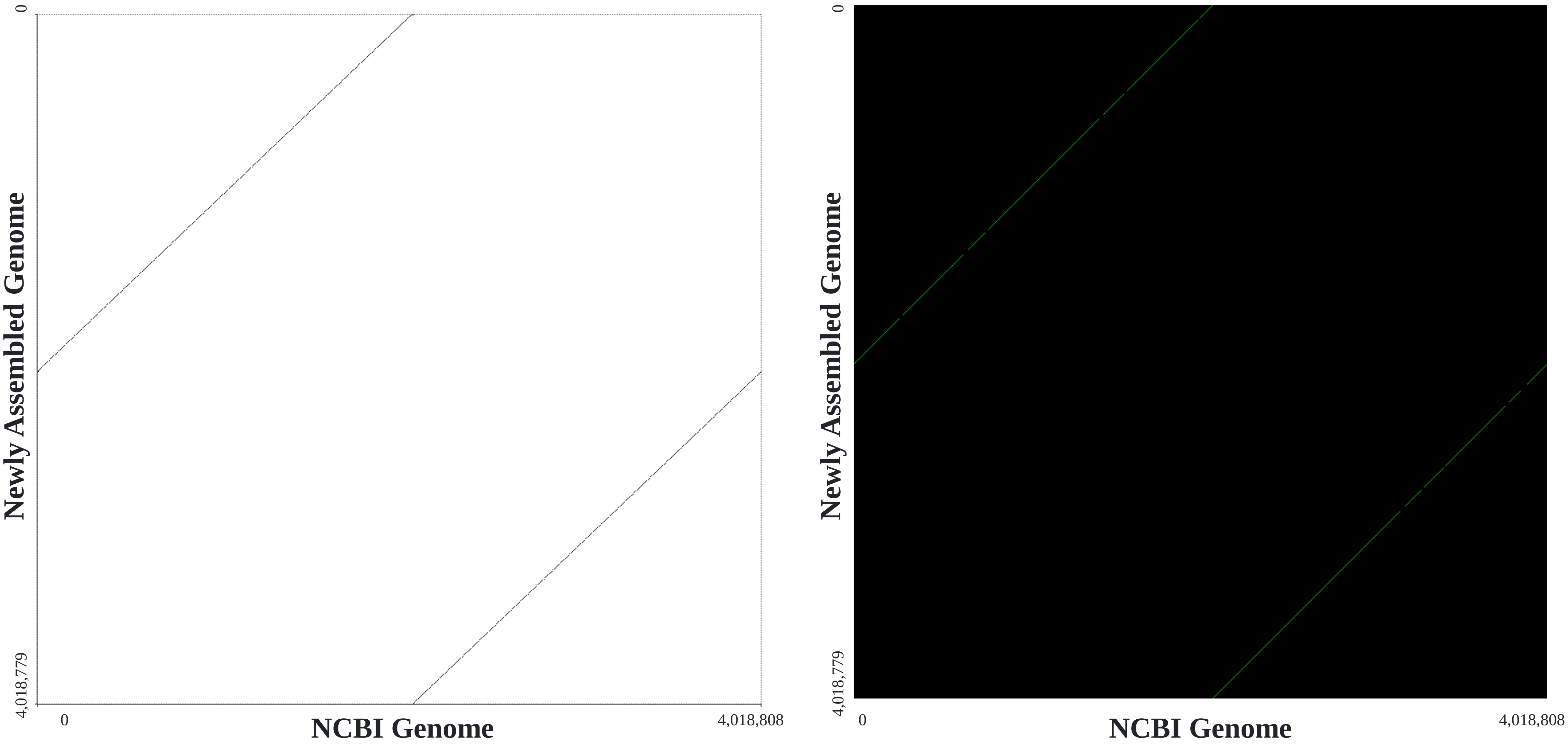

Figure S1. Alignment of Genome using Chromeister
Chromeister showed conserved blocks (red), inverted blocks (green), and transposed blocks (blue).
