# Supplementary material for: Genome-Wide Pathway Exploration of the Epidermidibacterium keratini EPI-7T
Source: Microorganisms. 2023 Mar 28;11(4):870. doi: 10.3390/microorganisms11040870 (PMC10143877; doi:10.3390/microorganisms11040870)
Supplement: Supplementary file 1 [file microorganisms-11-00870-s001.zip › Figure S2.pdf]

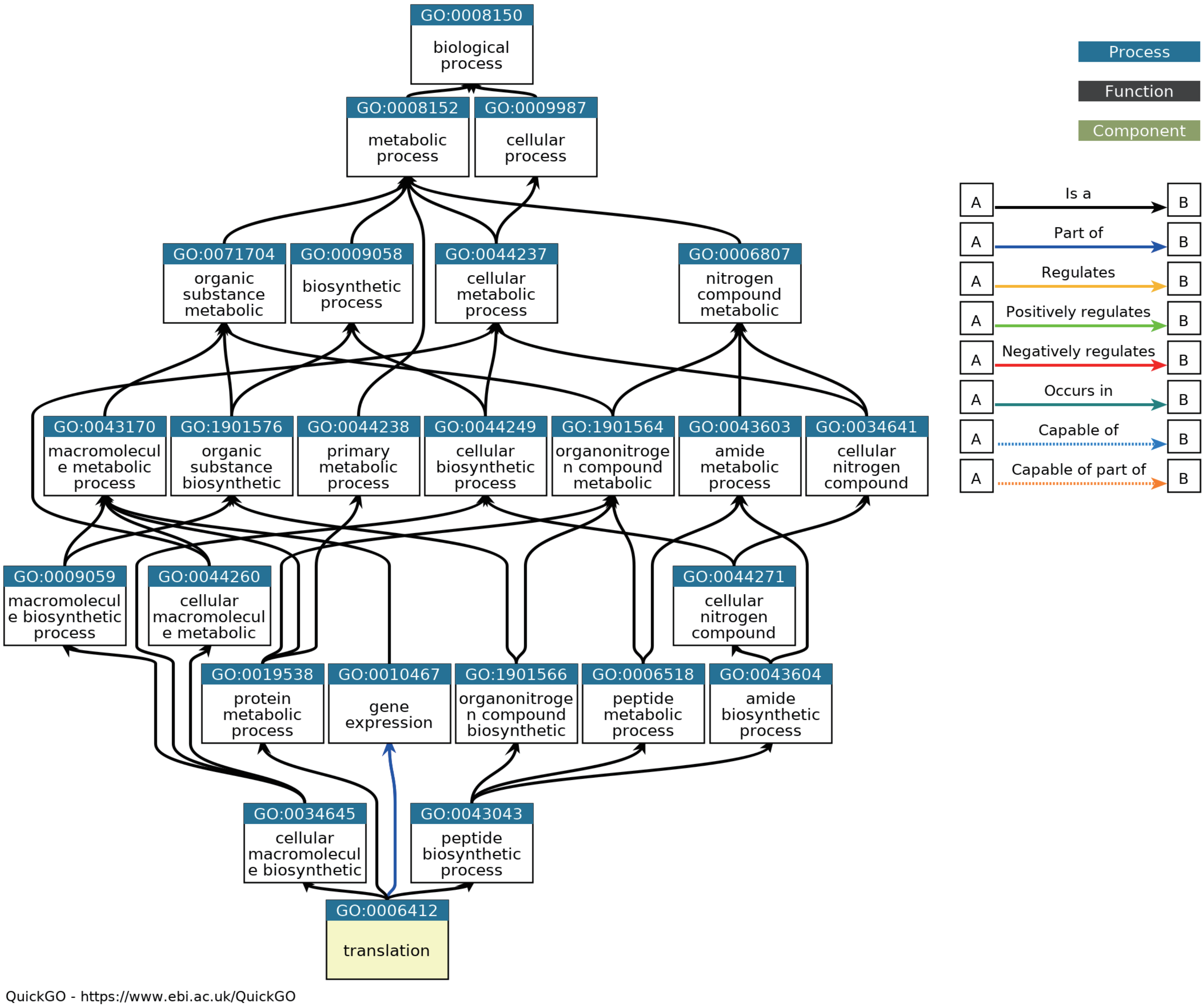

Figure S2. Summary of shared GO enrichment between *E. keratini* EPI-7^{T} and seven skin flora.
The results are summarized in GO enrichment shared by skin flora and *E. keratini* EPI-7^{T}. Three main categories: cellular components (green), molecular function (grey), and biological process (blue). And then, in GO enrichment, GO categories and GO terms of the clusters shared by the seven comparison strains . Translation" (GO:0006412, 46 clusters); "SOS response" (GO:0009432, 8 clusters); "'de novo' IMP biosynthetic process" (GO:0006189, 6 clusters); "Translation elongation factor activ ity " (GO:0003746, 4 clusters); "Translation initiation factor activity" (GO:0003743, 3 clusters) were shown significant associations.

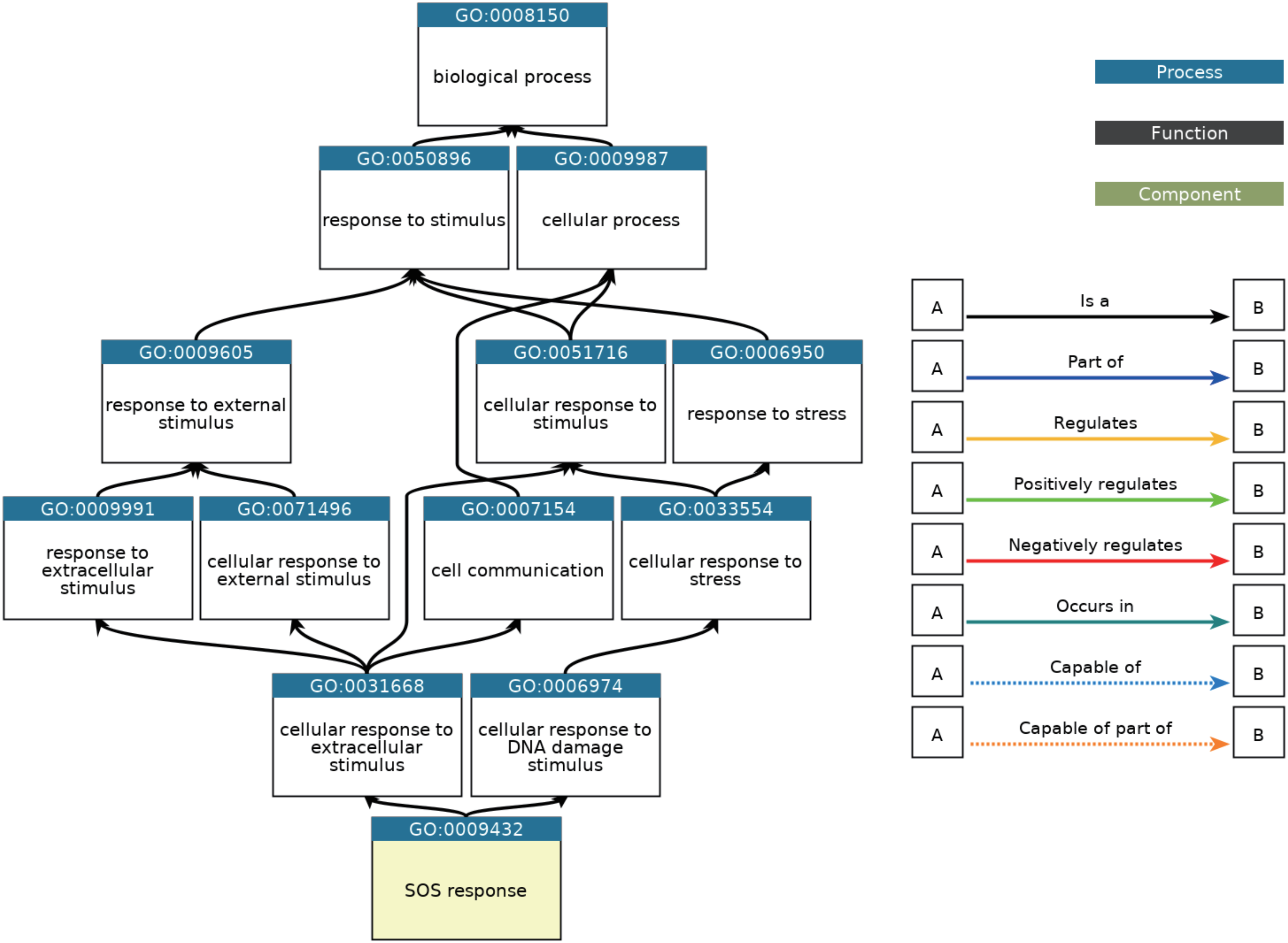

QuickGO - https://www.ebi.ac.uk/QuickGO

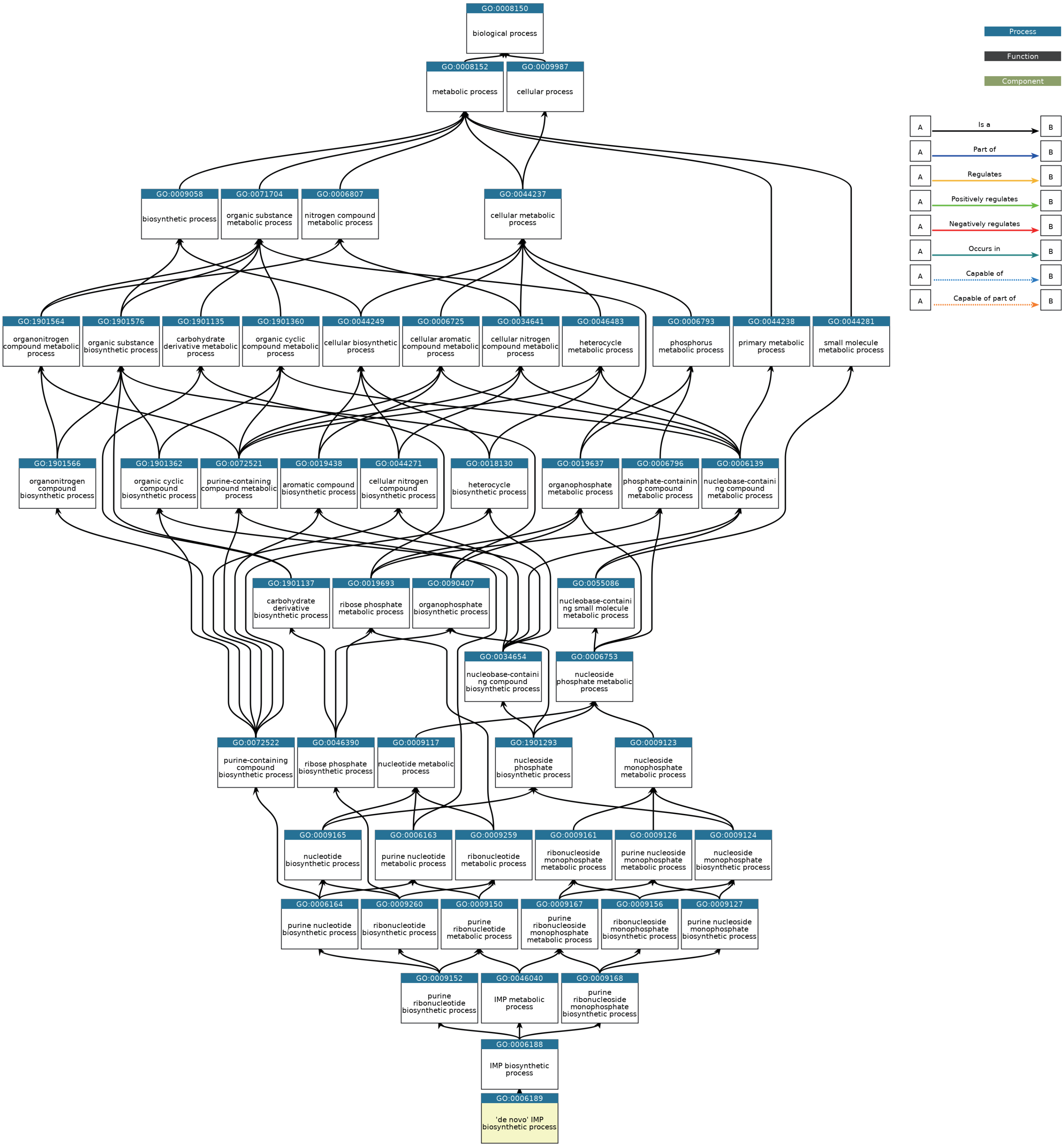

QuickGO - https://www.ebi.ac.uk/QuickGO

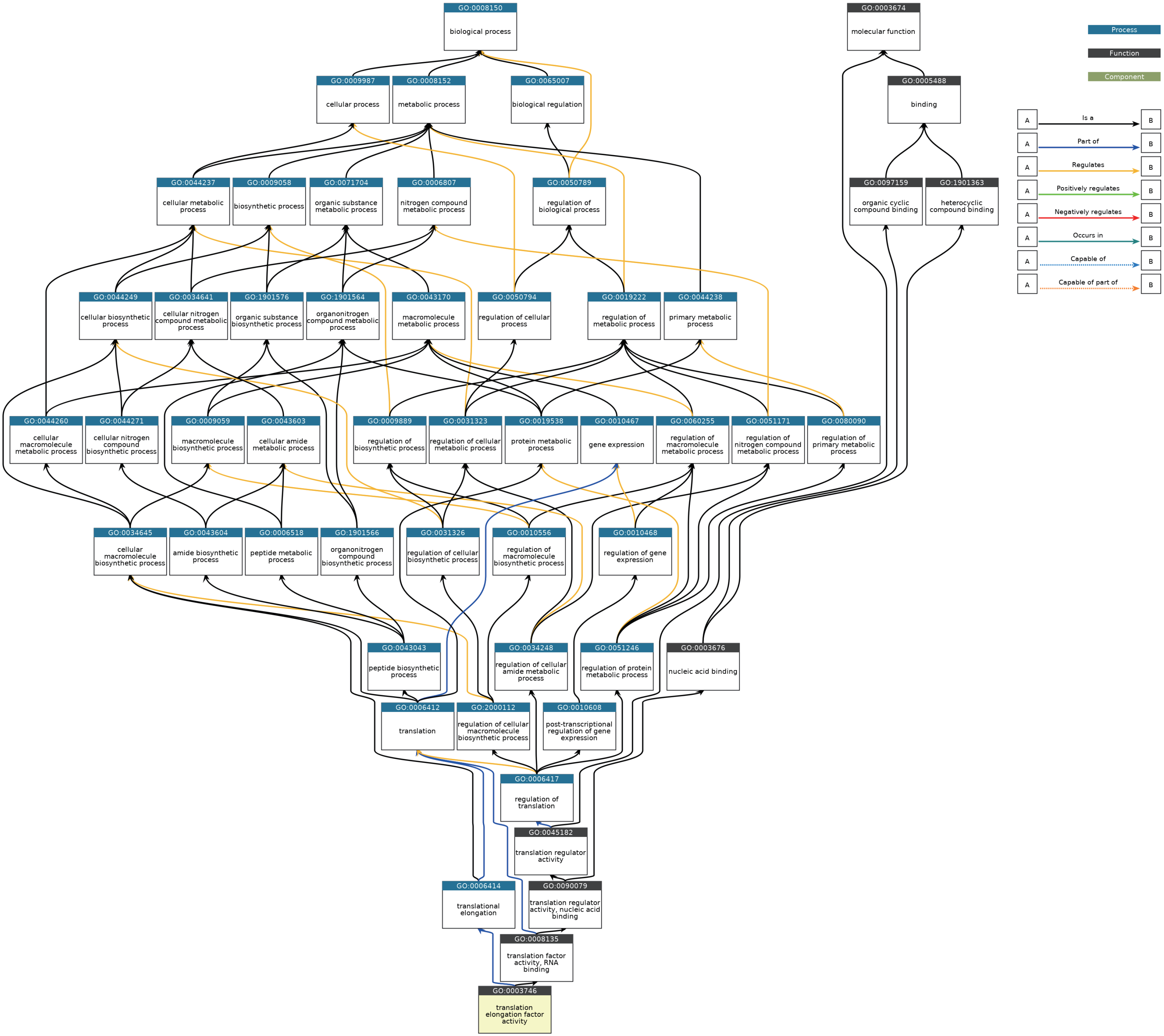

QuickGO - https://www.ebi.ac.uk/QuickGO

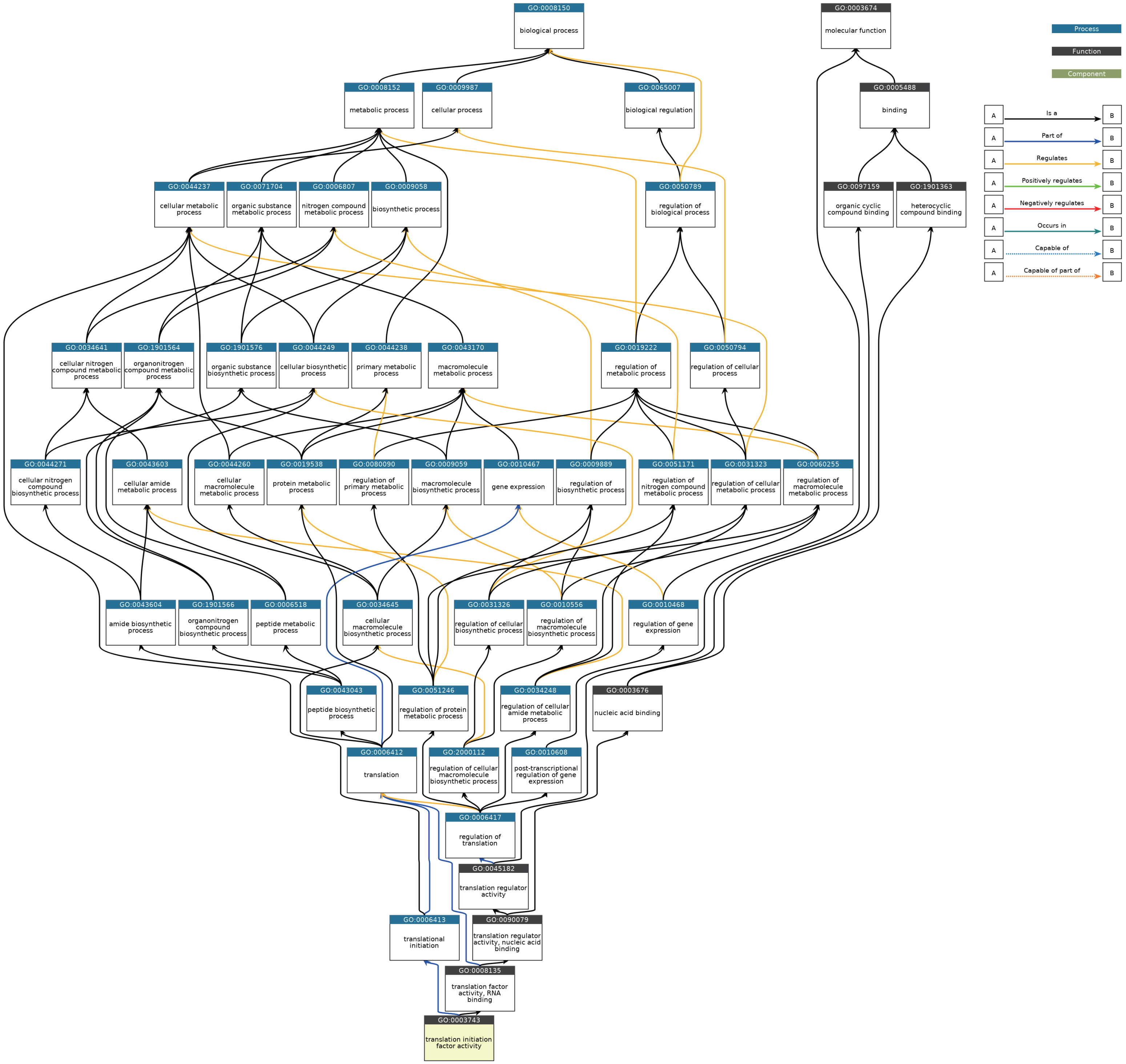

QuickGO - https://www.ebi.ac.uk/QuickGO
